# Recent decline in vegetative regeneration of bamboo (*Yushania alpina*), a key food plant for primates in Volcanoes National Park, Rwanda

**DOI:** 10.1038/s41598-019-49519-w

**Published:** 2019-09-10

**Authors:** Yntze van der Hoek, Faida Emmanuel, Winnie Eckardt, Innocent Kwizera, Mia Derhé, Damien Caillaud, Tara S. Stoinski, Deogratias Tuyisingize

**Affiliations:** 1 The Dian Fossey Gorilla Fund International, Musanze, Rwanda; 2 Department of Anthropology, The University of California, Davis, USA

**Keywords:** Forest ecology, Tropical ecology, Plant ecology, Conservation biology, Tropical ecology

## Abstract

The African montane bamboo *Yushania alpina* provides both habitat and food for many species in the Albertine Rift region. In Volcanoes National Park (VNP), Rwanda, it is especially important as a key food resource for the Endangered mountain gorilla *Gorilla beringei beringei* and Endangered golden guenon *Cercopithecus mitis kandti*. We examined temporal and spatial variation in bamboo shoots regeneration and consumption by primates, monitored between 2013 and 2018 in 82 16-m^2^ plots located along transects in VNP. Our analyses revealed a decline in vegetative regeneration of bamboo in recent years, which is mirrored by a decline in bamboo shoot consumption by primates; but an increase in proportional intake. Local declines in regeneration are potentially due to high intensities of herbivory, decreased amounts of rainfall during growing seasons, and natural processes that form part of the life cycle of bamboo. Moreover, spatial variation in bamboo regeneration can be explained by elevation, as well as by stand-level variation in soil acidity, vegetation density, and the density of dead bamboo culms. We discuss the potential mechanisms underlying observed temporal and spatial variations and outline possible effects of a decline in bamboo regeneration for primates and other aspects of biodiversity in VNP.

## Introduction

Bamboo forests occur in most tropical regions and serve as habitat for a large variety of species. In addition, bamboo shoots, and leaves and stems of mature bamboo, are sought after by both herbivores and people^1^. However, there are uncertainties regarding the future of bamboo forests, due to the direct and indirect impacts of anthropogenic activities, such as harvesting^2^ and climate change^3,4^, on bamboo regeneration and bamboo stand longevity.

The African montane bamboo *Yushania alpina* (K. Schum.) W.C. Lin. (1974), previously known as *Arundinaria alpina* and *Sinarundinaria alpina* and hereafter also referred to as ‘bamboo’, is widely distributed across the mountains of Central and East Africa. It is commonly found on volcanic soils at high elevations, predominantly between 2,600–3,000 m, where it often dominates the vegetation, resulting in monodominant ‘bamboo zones’^5^. The vegetative and sexual regeneration of *Y*. *alpina* typically involves cyclic processes^6–8^. Shoots tend to emerge from rhizomes every few years, especially in periods coinciding or directly succeeding long rains, and flowering cycles occur at intervals ranging from 15 years to 40 years^2,9^. The entire growth cycle of *Y*. *alpina* might last even longer^7–9^, as rhizomes might persist for decades before shoots start to form^9^. *Y*. *alpina* is monocarpic, and death of entire sections of bamboo forest will usually follow synchronized flowering and seed-forming events. It is unclear how bamboo forests regenerate after such events, or to what extent gap-creating disturbances such as fire play a role in these processes^10,11^, but bamboo might regenerate from seeds and from surviving rhizomes of old bamboo stands^12^. Outside of flowering events, the death of individual culms provides conditions that promote sprouting, likely similar to compensatory regrowth after herbivory^13^. In forests with little or no human disturbance, the death of culms and their replacement by new shoots occurs at roughly equal rates^9^.

Bamboo zones in the Albertine Rift region of East Africa provide habitat to species such as Archer’s robin-chat *Cossypha archeri* and Rwenzori apalis *Apalis ruwenzorii*, two birds particularly associated with the open understory found in bamboo forests^14^. In addition, bamboo is a food resource for various African vertebrates, especially the Endangered mountain gorilla *Gorilla beringei beringei* and the Endangered golden guenon *Cercopithecus mitis kandti*^15–17^, also known as golden monkey. Bamboo can make up a considerable portion of the diet of these primates in the Virunga massif (central Albertine Rift region)—up to 15% and 60% of the annual feeding time of mountain gorillas and golden guenons is spend consuming bamboo, respectively^15,17–20^—and other large herbivores such as buffalo, elephant and antelopes feed opportunistically on bamboo shoots when available^21^.

Volcanoes National Park (VNP) in Rwanda constitutes part of the Virunga massif, a mountain range in the Albertine Rift region. Extensive bamboo zones are located near the boundary of the park, at elevations of approximately 2,500–3,000 m a.s.l.^21^. No previous studies of bamboo ecology exist for VNP, but we can gain some insights from studies conducted in the neighbouring Mgahinga Gorilla National Park (MGNP) in Uganda. Here, flowering events were observed in patches of a few hectares in the 1980s^22^, though current bamboo shoot regeneration in the region seems to result mainly from vegetative reproduction from rhizomes. Most bamboo stands in MGNP seemed to be in a building phase (see Agnew^8^ for an explanation of classifications of stages in the life cycle of bamboo) around eight years ago (~in 2010), as the diameter of young culms surpassed that of old culms^2^. Canopies tend to become increasingly dense and closed during this building phase, which usually promotes a shift in understory vegetation from pioneer species to shade-tolerant plants, and eventually leads to changes in abiotic factors such as a decline in soil pH^8^.

Interestingly, Sheil *et al*.^2^ mention that a suspected decline in bamboo regeneration was the main incentive to conduct their study of bamboo ecology in MGNP. In recent years, park staff of VNP as well as researchers affiliated with The Dian Fossey Gorilla Fund’s Karisoke Research Center hinted at similar trends in VNP. Such declines, if confirmed, could lead to limitations in food availability for species, such as gorillas and golden guenons. In addition, it could affect various aspects of the ecology of these primates, including reproductive behaviour, and could have consequences for a range of other biota (e.g., birds) associated with bamboo habitat.

A lack of bamboo shoot regeneration may be driven by many factors. It could indicate that bamboo stands in the region underwent natural succession towards a mature phase, during which there is no increase in culm height or diameter^8^. It has been suggested that there is a decrease in shoot development near the end of this mature phase, before the onset of flowering (ref.^23^ cited in ref.^2^). In addition, lack of bamboo sprouting could result from high levels of herbivory^24^. Current knowledge of gorilla^25^ and golden guenon^17^ feeding ecology suggests that both species are unlikely to cause systematic mortality of their main food plants, but localized resource depression has been observed^15^ and patterns of resource use could change under increased population densities of both primates. Alternatively, herbivory, as well as trampling by other large herbivores, such as elephant and buffalo, could have an opposite effect and result in increases in shoot development through compensatory regrowth^25–27^—thus indicating that bamboo regeneration could actually be limited by a lack of large herbivores. Additionally, there are indications that bamboo competes for resources with other plants species, especially lianas^28^. These plants, in turn, are also consumed by large herbivores, thus indirectly affecting bamboo growth. Finally, bamboo regeneration could be affected by human-induced disturbances and stress, especially harvesting and climate change, or potentially even by indirect alterations such as changes in gap-creating wildfire regimes^11^. We know that human use can have both positive and negative effects on bamboo regeneration^2,29^, but the effects of climate change on *Y*. *alpina* have not been studied at all. However, we know that VNP experiences changes in both temperature and rainfall^30^, important determinants of the distribution of *Y*. *alpina*^27,31,32^, and thus we do predict effects of climate change on *Y*. *alpina* regeneration, similar to the declines in regeneration following climate change seen in other bamboo species^3,4^.

**Figure 1:**
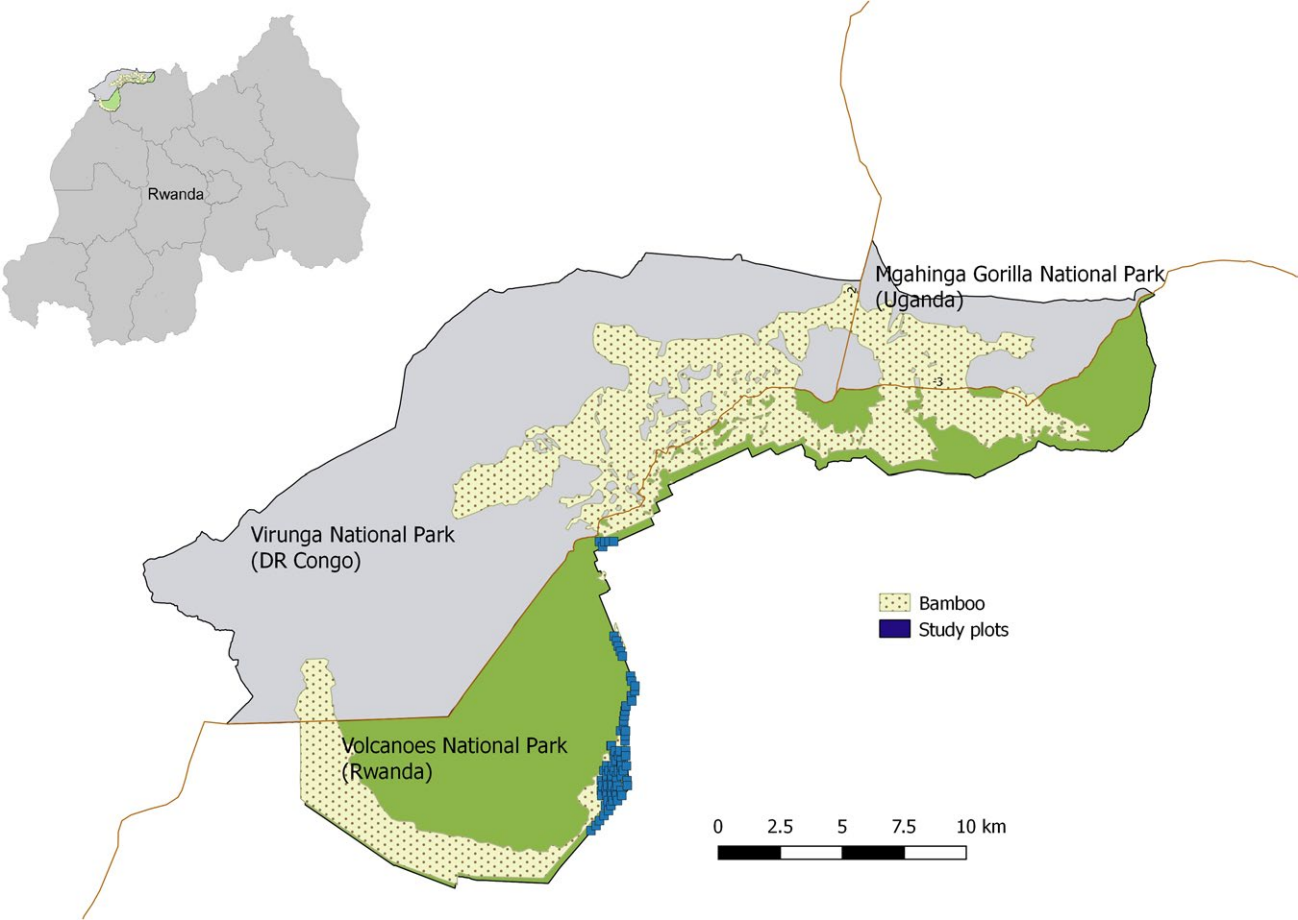
Map of the study area and location of plots in Volcanoes National Park, Rwanda.

With this study, we aim to improve our understanding of bamboo ecology in VNP in order to inform conservation and management of bamboo-associated species. In VNP, bamboo shoots emerge during two distinct seasons (hereafter the early and late growing seasons), following the onset of rainy seasons. Although long-term changes in availability of key gorilla food plants was the focus of previous studies in VNP, no analyses of temporal trends in bamboo shoot availability are currently available^20^. Here, we address whether there were any trends in bamboo shoot regeneration in selected plots in VNP (Fig. 1) over a 6-year period, from 2013 to 2018. In addition, we aimed to determine whether trends in bamboo shoot regeneration were linked to bamboo consumption by primates and explored which bioclimatic and environmental factors influence both temporal and spatial variation in bamboo shoot regeneration.

## Results

### Temporal, spatial, and climatic effects on bamboo regeneration and consumption

**Figure 2:**
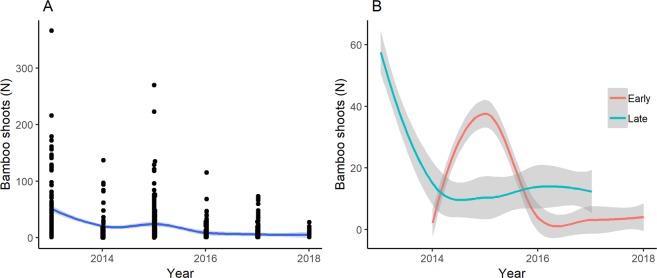
Bamboo shoot regeneration over the years 2013–2018. LOESS plots of the average number of bamboo shoots over the years (**A**) and of those bamboo shoots found in the late (September–December) and early (March–May) growing seasons only (**B**). Points in A show numbers of bamboo shoots found in our plots.

Between 2013 and 2018, we found a significant (p < 0.001), 83-percent decline in the number of newly regenerated shoots, a trend that was especially pronounced for the late growing season (GAMM; Fig. 2; Table 1). The largest portion of this decline occurred before 2015, with only a minor decline in bamboo shoot regeneration in recent years (2016–2018). This decrease in bamboo shoot regeneration was reflected by a significant decline in the absolute number shoots consumed by primates over the years (p < 0.001; from 9.26 on average in a plot in 2013 to 2.33 in 2018), and a significant increase in proportional consumption (p < 0.001; from an average of 17% of shoots consumed in 2013 to 38% consumed in 2018).

**Table 1:** Results of Generalized Additive Mixed Models (GAMMs) that approximate temporal trends in shoot regeneration (negative binomial), consumption (negative binomial), proportional consumption (quasibinomial) and growth rates (loglinear Gaussian).

Response variable	Deviance explained (%)	Type of predictor variable	Variable	Estimate	SE	t value	P-value
Shoot regeneration	11.6	Linear	Season	20.090	3.529	5.687	<0.001
			Elevation	0.005	0.001	5.539	<0.001
			Season * Elevation	−0.007	0.001	−5.420	<0.001
					*edf*	*F*	
		Smoother	Year		4.938	32.61	<0.001
Shoot consumption	8.3	Linear	Season	15.223	3.615	4.211	<0.001
			Elevation	0.003	0.001	3.417	<0.001
			Season * Elevation	−0.005	0.001	−4.046	<0.001
					*edf*	*F*	
		Smoother	Year		4.514	11.03	<0.001
Proportional shoot consumption	13.3				*edf*	*F*	*P-value*
		Smoother	Year		4.342	15.88	<0.001
Growth rates	19.2	Linear	Season	0.688	0.093	7.365	<0.001
			Elevation	−0.002	<0.001	−3.385	<0.001
					*edf*	*F*	*P-value*
		Smoother	Year		2.895	25.28	<0.001

edf = effective degrees of freedom.

We found a significant difference between regeneration in the two growing seasons (GAMM; p < 0.001; Fig. 2B, Tables 1 and 2), with overall levels of regeneration being lower in the early as compared to the late growing season. There was also a significant increase in shoot regeneration with elevation (p < 0.001), though this was predominantly caused by relatively high shoot regeneration at high elevations in the early growing season and was not observed for the late growing season (evidenced by a significant interaction effect between season and elevation; p < 0.001). Overall consumption was significantly higher in the late as compared to the early growing season (p < 0.001), and again higher at high elevations (p < 0.001), at least in the early growing season (interaction effect season x elevation; p < 0.001). Despite differences in overall consumption, we found no significant difference between the seasons in the proportional consumption of shoots (p = 0.760). Specifically, we found that both bamboo shoot regeneration and consumption in the early growing season was higher at high elevations than at low elevations, whereas the reverse was true for the late growing season. Finally, we found a significant increase in the growth rate of bamboo shoots over the years (p < 0.001) and a negative effect of elevation on growth rate (p < 0.001), but no differences in growth rates between the growing seasons (p = 0.877).

**Table 2:** Mean bamboo shoot regeneration (numbers), consumption (numbers and proportions), and growth rates (cm/day) in Volcanoes National Park (Rwanda) in two distinct growing seasons and at two different elevations (SD in parentheses) between 2013–2018. The relatively high SDs reflect skewed and non-normal distributions.

	Variable	Low Elevation	High Elevation
Early growing season	Number of shoots Number of consumed shoots Proportion of shoots consumed Shoot growth rate in cm/day	7.2 (22.5) 2.2 (4.9) 0.23 (0.32) 9.5 (6.6)	12.8 (25.9) 3.3 (4.7) 0.35 (0.35) 8.0 (7.3)
Late growing season	Number of shoots Number of consumed shoots Proportion of shoots consumed Shoot growth rate in cm/day	23.4 (36.3) 6.1 (9.6) 0.30 (0.33) 7.4 (6.4)	19.4 (36.3) 4.2 (5.6) 0.33 (0.34) 6.4 (7.4)

The decline we found in bamboo shoot regeneration mirrored the significant (GAMM; p < 0.001; Table 3) decline in the amount of rainfall during bamboo growing seasons over the 2013–2018 period, from an average of ~182 mm (late 2013 / early 2014) to ~121 mm (late 2017/early 2018), with no significant effect of season (p = 0.115). In contrast, we found that minimum/maximum temperatures increased from an average of 11.8/23.4 °C (calculated over late 2013 and early 2014) to 13.1/23.7 °C (late 2017 and early 2018), though these increases in temperatures were not significant (p > 0.05 for both minimum and maximum temperatures).

**Table 3:** Results of three separate Generalized Additive Mixed Models (GAMMs) with Gaussian error structures that approximate temporal trends in rainfall (mm) and minimum and maximum temperatures between 2013 and 2018 near Volcanoes National Park, Rwanda. Year was the predictor variable in all three models.

Response variable	Deviance explained (%)	*F*	*edf*	*P-value*
Rainfall	49.0	5.13	2.366	<0.001
Maximum temperature	53.5	3.619	2.862	0.070
Minimum temperature	11.7	3.35	1	0.104

edf = effective degrees of freedom.

### Relationships between bamboo shoot regeneration and consumption

**Figure 3:**
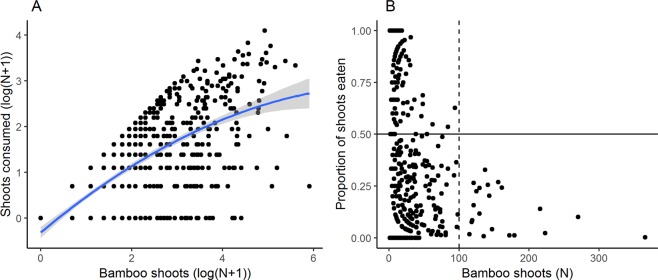
Relationships between number of bamboo shoots available in a plot and consumption by primates. Panel (A) shows the number of shoots consumed by primates versus bamboo shoot availability, with the blue line and grey band representing a quadratic relationship and ±95% CI. Panel (B) shows primates never consumed more than half of shoots consumed in plots with particularly high numbers of shoots (>100).

**Figure 4:**
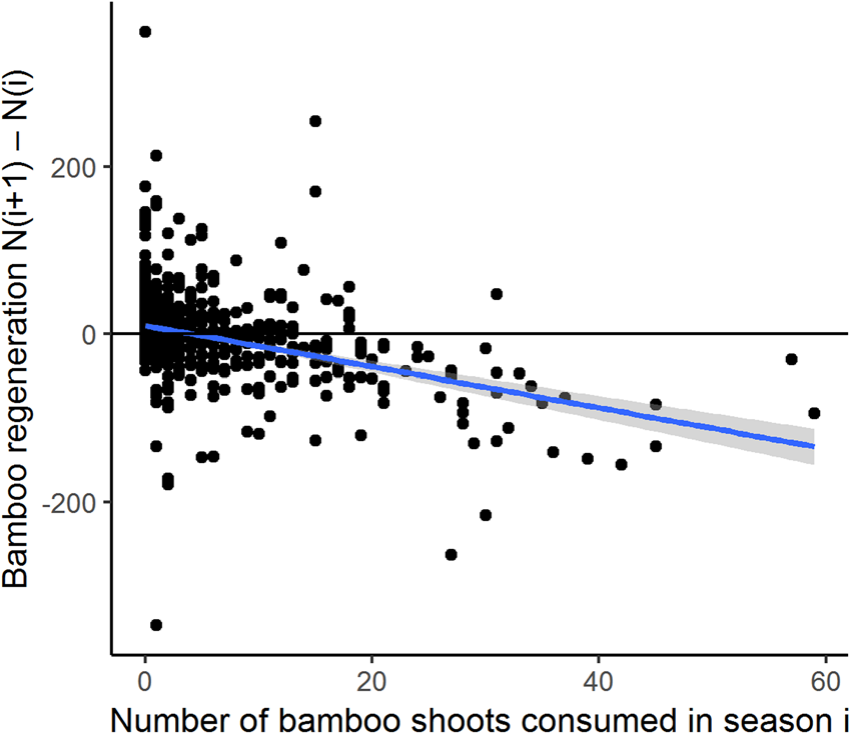
The relationship between primate herbivory and bamboo shoot regeneration. Depicted is the relationship between the number of bamboo shoots consumed in a survey plot in a given season (i) and the difference in the number of bamboo shoots (as compared to the current season) that emerge a season later (N(i + 1)-N(i)). Blue line and grey band represent a linear relationship and ±95% CI.

There was a positive relationship between the number of bamboo shoots that emerged in a given growing season and the number of bamboo shoots consumed that same season (GLM; b = 0.022, SE = 0.001, t-value = 23.968, p < 0.001; Fig. 3A). However, primates did not seem to increase their intake linearly in proportion to availability. If anything, there seemed to be proportionally less consumption in plots with particularly high (100 shoots or more) numbers of young bamboo shoots (GLM; b = −0.005, SE = 0.002, t-value = −2.150, p = 0.032; Fig. 3B), and hardly ever were more than 50 shoots consumed in any one season in any one plot. Finally, we found a negative relationship between bamboo shoot consumption by primates in one given growing season and bamboo shoot regeneration in the next growing season (GAMM; b = −2.266, SE = 0.198, t-value = −11.426, p* < *0.001; Fig. 4).

### Environmental correlates of bamboo shoot regeneration

We found that spatial patterns in bamboo shoot regeneration between 2013 and 2018 were potentially (co-)determined by at least three environmental variables (GLM; p < 0.05; Table 4). Specifically, bamboo shoot regeneration was negatively related to soil pH (p = 0.016) but increased with higher vertical understory densities (p < 0.001) and numbers of dead bamboo culms (p = 0.006) found in a plot.

**Table 4:** The effect of environmental factors on spatial variation in bamboo shoot regeneration within 82 survey plots in Volcanoes National Park, Rwanda, based on outputs from a negative binomial Generalized Linear Mixed Model (GLMM).

Random effect			Variance	Std. Dev.
Transect			0.061	0.248
**Fixed effects**	**Estimate**	**SE**	**t value**	**P-value**
Soil pH	−0.265	0.115	−2.231	0.026
Vertical understory density (%)	0.009	0.004	2.511	0.012
Number of dead bamboo culms	0.064	0.030	2.154	0.031

## Discussion

Our aim was to test for temporal trends in bamboo shoot availability in Volcanoes National Park (VNP), Rwanda over recent years. We show that, between 2013 and 2018, there was a decline in the number of bamboo shoots that emerged through vegetative regeneration. Although most of this decline occurred before 2015, current rates of bamboo shoot regeneration are at such low levels that even small future declines could eventually lead to a complete lack of bamboo shoot regeneration in our study plots. Moreover, we provide evidence that this decline in bamboo shoot availability is mirrored by a decline in the consumption of shoots by primates, and by an increase in proportional consumption. If proportional consumption increases further, then competition for bamboo shoots as a food resource might rise accordingly.

Trends in climatic conditions (e.g., VNP has seen a decline in rainfall and increase in temperatures, this paper), increases in primate population sizes which may have led to an increased intensity of herbivory, and natural succession of bamboo stands are all factors that possibly contribute to observed declines in bamboo shoot regeneration. Though it is difficult, for now, to separate causative from correlative relationships, we briefly discuss these potential causes of declines in shoot regeneration. Regarding climatic conditions, we note that *Y*. *alpina* generally grows in regions where annual rainfall is between 800 and 2,000 mm and mean annual temperature ranges from 10 to 20 °C^27,31,33^. Although the current climate in VNP approaches such climatic conditions (minimum and maximum annual temperature 12–24 °C at lower elevations of the park and ca. 1,000 mm rain annually), we did find that rainfall during the growing seasons has declined in recent years, whereas temperatures have increased. Following these trends in climatic conditions, we could tentatively argue that abiotic conditions become increasingly less favourable at lower elevations, and more favourable at higher elevation, which could in turn explain the observed higher rates of regeneration at higher elevations. We propose that locally obtained microclimatic data (i.e., as measured within plots and across elevations) is incorporated in future explorations of the relationships between trends in climatic conditions and bamboo shoot regeneration. Climatic data with such ‘high spatial resolution’ will also allow us to explore possible effects of changes in rainfall seasonality and possible elevational range shifts for bamboo and associated biota^34^.

Effects of increased herbivory may also have contributed to observed temporal patterns of declines in bamboo shoot regeneration. For one, we found evidence of an increase in proportional consumption of bamboo shoots over recent years, as well as a relationship between the availability of bamboo shoots in a given growing season and consumption by herbivores, though possibly with an upper limit to the amount of bamboo shoots consumed at one particular location at a given time. This relationship confirms the importance of bamboo shoots as a food resource among primates in VNP but also leads to questions regarding possible effects of increases in competition for bamboo shoots, for example as a result of growing gorilla populations^35^. In this context, we also point to signals that high levels of herbivory may add to local declines in bamboo shoot regeneration in a subsequent season—which could reflect a form of resource depression also observed by Watts^15^—a topic for additional experimental studies.

Finally, we consider the role of natural processes in the bamboo life cycle and succession of bamboo stands. Interestingly, the growth rate of bamboo shoots increased over recent years. This is possibly due to altered rates of photosynthesis linked to climate change^36^, but may also indicate that bamboo stands have reached a ‘building phase’ in which we see an increase in the diameters and heights of subsequent generations of regenerated bamboo culms^8^. Nevertheless, given the fact that the complete reproductive cycle of *Y*. *alpina* (from seed germination and seedling recruitment after a mass flowering event to death of a bamboo stand) takes anywhere from 15 to 40 years to complete, we deem it likely that at least some of the bamboo stands in VNP have reached the mature phase^8^, when there is no further increase in bamboo size, with a lack of regeneration at some locations as a result.

With regards to spatial variation in bamboo shoot regeneration, we found relatively high rates of shoot regeneration in dense bamboo stands with large numbers of dead bamboo culms. High rates of shoot regeneration may result from altered understory conditions (e.g., sunlight reaching the forest floor^10^) or from compensatory regrowth after the death of bamboo culms and the availability of high densities of rhizomes from which new shoots might emerge. In addition, our finding that shoots regenerate more frequently at locations with a relatively low soil pH may, with caution, be linked to increased overall bamboo productivity on such soils^37^. Alternatively, the pH of soils in close vicinity to bamboo stands is known to decline as bamboo stands mature^8^, and relatively high rates of bamboo regeneration on more acidic soils can thus also potentially be linked differences in the age of bamboo in different stands—which may itself influence stand-level characteristics such as rhizome densities. More experimental approaches (e.g., changing soil characteristics within certain plots) may clarify the links between environmental correlates and spatial patterns in bamboo regeneration, as well as explore the possible effects of disturbances, such as soil trampling, bamboo harvesting, and fire^11,25–27,29,38^.

Regardless of the cause of the observed decline of bamboo regeneration, we may briefly address possible consequences of a potential lack of shoot availability for some of the animals that use it as a food or habitat resource. With regards to the mountain gorillas of VNP, we deem it probable that mountain gorillas can show some dietary flexibility^20^ if bamboo shoots become scarce, especially as it is clear that bamboo does not form a substantial part of the diet of all gorillas in VNP^15^. On the other hand, although there is no current direct evidence of food competition^39^, a decrease in bamboo shoot regeneration might become a problem if the population of mountain gorillas in VNP continues to increase. Besides, even if gorillas are not physically limited by a lack of bamboo shoot availability, there might be indirect consequences that are mediated through changes in behaviour. For example, the activity budget and energy expenditure of individual gorillas can change considerably as a result of the seasonal increase in bamboo shoot consumption^40^, and a lack of bamboo shoot availability can have unknown consequences for various gorilla behaviours, such as play and processes of weaning.

A lack of bamboo shoot availability might also not directly affect the nutrient uptake by individual golden guenons, as a large portion of their diet is made up of parts of mature bamboo (especially leaves) in addition to the occasional intake of other food items (fruits, flowers, leaves of other plants, insects)^17,41^. However, a lack of bamboo shoot regeneration is likely to impact golden guenon behaviour and population dynamics. For example, it may induce shifts in home-ranges or changes in reproductive patterns, especially as current birthing seasons seem linked to bamboo growing seasons^17^. Finally, we note that additional research will be needed to determine whether, and to what extent, a lack of bamboo availability leads to an increase in crop-raiding by primates.

Diminished vegetative regeneration, or even death of certain bamboo stands, does not have to be a worrisome development with only negative consequences, as it may be part of a natural succession cycle. For example, as bamboo mass-flowering and die-off events may increase the survivorship and growth of saplings of overstory tree species^42^, we could hypothesize that mass die-off of bamboo in VNP may have positive effects on the regeneration of the keystone tree *Hagenia abyssinica*^43^. Moreover, it is unlikely that bamboo will die-off instantaneously across VNP and both of the main consumers of bamboo (mountain gorillas and golden guenons) are likely to show some dietary flexibility. Moreover, although we found significant declines in bamboo regeneration in our study plots, it should be noted that 82 survey plots represent only a small portion of all the bamboo forest found in the Virunga Massif. Bamboo stands across the Virunga Massif, including VNP, show spatial variation in age, culm density and a host of other variables, and we consider it highly unlikely that bamboo regeneration will come to a simultaneous halt across the entire region. Nevertheless, we deem it important that conservation and research efforts focus on continuous monitoring of bamboo regeneration, in order to detect further changes. Specifically, there is a need for continuous monitoring of the responses of consumers of bamboo shoots to local decline/absences of this resource. Such research is urgent, as both mountain gorillas and golden guenons are endangered and occur in small populations in a relatively small and isolated geographic region, which could limit these primates’ opportunities to adapt to changes in food availability.

## Methods

### Study area and data collection

We studied vegetative regeneration of *Yushania alpina* in Volcanoes National Park (VNP) in north-western Rwanda, located between 1°21′–1°35′S and 29°22′–29°44′E (Fig. 1). Between September 2013 and June 2018, we collected data in 82 plots placed along 21 transects that were set up to cover an elevational range from 2,610 to 3,040 m a.s.l. Transects were placed approximately 200 m apart, to reduce spatial autocorrelation among our data, and varied in length (400–1,600 m) according to the width (as measured along the slope of the mountains) of the bamboo zone. Along each transect, we placed 4 m × 4 m plots at 200 m intervals. During the study, we visited each plot biweekly during the course of two distinct bamboo shooting seasons (the short (March–May) and long rainy season (September–December), hereafter called the early and late growing seasons, respectively). In each plot, we counted all bamboo shoots, marked them with a unique code, measured their heights, and recorded whether they were fed on by primates (gorillas and/or golden guenons). We concluded there was consumption by primates when we identified particular bite marks on bamboo shoots in combination with other signs (e.g., evidence of shoot pealing or foot prints). To ensure accurate identification of consumption, we drew from our collective—as an organization—extensive history studying primate feeding behaviour in the field^18^.

The Rwanda Meteorology Agency provided climatic data, specifically monthly rainfall (mm), and maximum and minimum temperatures for the years 2013–2018, from a weather station in the town of Musanze (1°29′S, 29°37′E, 1878 m a.s.l.) approximately 8–10 km from the nearest study plot and 732 metres lower than the lowest of our 82 study plots. During one site visit, in 2017, we measured various additional environmental variables in each plot. First, we looked at topographic factors known to influence the growth of bamboo, at least that of other species^44,45^. We calculated slope steepness (in percent) at each plot using a person-to-person method and a clinometer. Steepness of slope is known to influence the growth and regeneration of other species of bamboo, as it is a proxy for both soil depth (*i*.*e*., levelled slopes will generally have deeper soil profiles than steep slopes) and reception of solar radiation^44^. Finally, we calculated the mean soil pH in each plot, by averaging measurements (using an Oakton pH 150 meter) of soil samples taken at two random locations within each plot.

We also measured vegetation characteristics in an extension (14 m × 14 m) of each plot. First, we used a fisheye hemispherical lens to photograph the canopy at each corner of an expanded plot, analysed these circular images with Gap Light Analyzer v. 2.0^46^, and calculated the mean canopy cover at each plot. Canopy cover is known to influence light conditions in bamboo forests, which subsequently influences bamboo shoot regeneration, at least in other bamboo species^45^. We calculated canopy height, another factor influencing light conditions, at each plot, by averaging two measurements taken with a clinometer at two sides of each plot. Light conditions at ground level might moreover be influenced by the density and structure of strata below the canopy^47^. We therefore estimated vertical vegetation structure of the understory shrub layer, which—being predominantly made up of bamboo—indirectly provided us with a proxy for bamboo culm density. For this, we photographed the understory shrub layer against a background consisting of a black sheet (1 m × 1 m) arranged perpendicularly to the ground. We used a normal 65 mm lens on a camera positioned approximately 3 m from the background, and took photographs at two corners of a plot, looking into the centre of the plot. From these photographs, we calculated vertical vegetation density using Sidelook v. 1.1^48^. Next, we estimated ground cover in two 50 cm × 50 cm quadrats placed 2 m from the centre point of each plot. We visually estimated the percentage of ground covered by vegetation in each of these quadrants. Finally, as vegetative regrowth of bamboo might be triggered by the death of bamboo culms, we counted the number of dead bamboo culms found in each plot.

### Data analyses

Our data analyses consisted of: (1) time-series analyses to assess trends in bamboo shoot regeneration, bamboo consumption by primates, and bamboo shoot growth rates; (2) regression modelling of the trends in climatic factors over the study period; (3) analyses of the relationship between primate herbivory and bamboo shoot regeneration; and 4) tests to assess potential relationships between environmental factors and patterns in bamboo shoot regeneration. We conducted all analyses using packages “lme4”^49^, “lmtest”^50^, “nlme”^51^, “tseries”^52^, “gam”^53^, “MASS”^54^, “mgcv”^55^ and base packages in R v. 3.5.0^56^.

To test for temporal trends in bamboo shoot regeneration and consumption, we first assessed temporal autocorrelation in our data. Initial data exploration using autocorrelation and partial autocorrelation function (ACF and PACF) plots revealed significant temporal autocorrelation between subsequent seasons. In addition, local regression analyses (LOESS) (Fig. 2) indicated that trends in bamboo shoot regeneration and bamboo shoot consumption over time were likely non-linear and that there were substantial differences between trends for the early and late growing seasons. Given these observations, we opted to fit generalized additive mixed models (GAMMs) to our data with an AR1 temporal autocorrelation term, which allow for effective modelling of nonlinear trends unlike alternative models such as seasonal autoregressive integrated moving average models (SARIMAs) (for similar uses see, *e*.*g*.^57–59^). Residuals of our AR1 models showed no significant autocorrelation.

We first fitted a full GAMM with a negative binomial error distribution, to handle overdispersion, to the number of newly developed shoots. As predictor variables we included year, elevation, and growing season, with a smoothing spline to capture possible non-linear effects of the variable year^58^. We also included an interaction term between elevation and growing season, given that field observations suggest differences in the regeneration rates at low and high elevations in the two seasons, and a corAR1 autocorrelation function to account for temporal correlations of measurements made within plots.

Next, we created GAMMs with the number of shoots consumed (negative binomial distribution to account for overdispersion), the proportion of shoots consumed (quasibinomial error distribution for proportions), and growth rates of the shoots (loglinear Gaussian error distribution, given positive skewness) as response variables. We again included year (smoother), elevation, and growing season as predictor variables and a temporal autocorrelation term (corAR1). We dropped non-significant terms from these full models before interpreting results. As our models indicated that trends in bamboo shoot regeneration differed between growing seasons and across elevations (see Results), we also provide summary statistics for both growing seasons and models for low and high elevations separately. For this, we split our data into a low and a high-elevation subset using the median elevation of our plots (2879 m a.s.l.) as cut-off.

To understand how climatic drivers might have changed over the years, we also looked at temporal trends in the average amount of rainfall (in mm) and maximum and minimum temperatures. We again used GAMMs (Gaussian error distributions after checks for normal distributions) with year and season as predictor variables, a smoothing spline for variable year, and a term to address temporal autocorrelation (corAR1). We decided not to explore statistical relationships between these climatic data and shoot regeneration and consumption variables as we did not have climatic data for the different plots or across elevations, but data from one single source at a relatively low elevation. In addition, we did not want to overqualify a possible similarity in temporal trends in climatic conditions and bamboo shoot regeneration, given we were unable to distinguish possible causative from correlative relationships.

We used a generalized linear model (GLM), with a negative binomial error distribution to address overdispersion, to test whether there was a relationship between the number of bamboo shoots that came up in a given growing season and the total number of bamboo shoots that showed signs of feeding by primates in the same time period. We repeated this exercise (with a quasibinomial error distribution for proportions) for the relationship between the bamboo shoot regeneration and the proportion of bamboo shoots consumed. Our next step was to test whether shoot consumption in a plot in one growing season had an effect on regeneration in the subsequent season. For this, we created a GAMM (Gaussian error distribution, normal distribution) with the number of shoots consumed as predictor variable, the change in bamboo shoot regeneration in the next season (i.e., the number of shoots, N_i+1_, produced in growing season i + 1 minus the number of shoots produced in the previous season, N_i_) as dependent variable, a corAR1 term to address temporal autocorrelation, and season as random effect.

Finally, we addressed possible links between a select number of environmental factors and spatial variation in bamboo shoot regeneration. For this, we created a generalized linear mixed model (GLMM; negative binomial error distribution) with the number of shoots that regenerated between 2013 and 2018 as the response variable. As predictor variables we included degree of slope (rescaled), soil pH (rescaled), vertical understory density (rescaled zero-mean and unit-variance), percentage of ground cover consisting of live vegetation (rescaled), canopy height (rescaled), and canopy cover (rescaled) of each plot. To address the effects of having plots nested within transects, and thus potentially less variation between plots within transects than between transects, we added transect as a random effect. We used variance inflation factors (VIFs) to test for collinearity among predictor variables that could potentially bias model outcomes and found no problematic correlations (VIFs were <2 for all variables, below the threshold at which collinearity becomes problematic^60^). From this full model we dropped non-significant terms before interpreting results.

## Supplementary information

Dataset 1

## Data Availability

Original raw data on bamboo shoot regeneration and consumption are available in Supplementary Dataset 1.
